# Effect of short-term smoking & L-arginine on coronary endothelial function assessed by cardiac magnetic resonance cold pressor testing: a pilot study

**DOI:** 10.1186/s12872-021-02050-1

**Published:** 2021-05-12

**Authors:** Andreas M. Weng, Herbert Köstler, Thorsten A. Bley, Christian O. Ritter

**Affiliations:** 1 Department of Diagnostic and Interventional Radiology, University Hospital of Würzburg, Oberdürrbacher Str. 6, 97080 Würzburg, Germany; 2 Institute for Diagnostic and Interventional Radiology, University Medicine Goettingen, Goettingen, Germany

**Keywords:** MRI, Myocardial perfusion, Cold pressor test, Endothelium, L-arginine, Smoking

## Abstract

**Background:**

The effect of smoking on coronary vasomotion has been investigated in the past with various imaging techniques in both short- and long-term smokers. Additionally, coronary vasomotion has been shown to be normalized in long-term smokers by L-Arginine acting as a substrate for NO synthase, revealing the coronary endothelium as the major site of abnormal vasomotor response. Aim of the prospective cohort study was to investigate coronary vasomotion of young healthy short-term smokers via magnetic resonance cold pressor test with and without the administration of L-Arginine and compare obtained results with the ones from nonsmokers.

**Methods:**

Myocardial blood flow (MBF) was quantified with first-pass perfusion MRI on a 1.5 T scanner in healthy short-term smokers (N = 10, age: 25.0 ± 2.8 years, 5.0 ± 2.9 pack years) and nonsmokers (N = 10, age: 34.3 ± 13.6) both at rest and during cold pressor test (CPT). Smokers underwent an additional examination after administration of L-Arginine within a median of 7 days of the naïve examination.

**Results:**

MBF at rest turned out to be 0.77 ± 0.30 (smokers with no L-Arginine; mean ± standard deviation), 0.66 ± 0.21 (smokers L-Arginine) and 0.84 ± 0.08 (nonsmokers). Values under CPT were 1.21 ± 0.42 (smokers no L-Arginine), 1.09 ± 0.35 (smokers L-Arginine) and 1.63 ± 0.33 (nonsmokers). In all groups, MBF was significantly increased under CPT compared to the corresponding rest examination (*p* < 0.05 in all cases). Additionally, MBF under CPT was significantly different between the smokers and the nonsmokers (*p* = 0.002). MBF at rest was significantly different between the smokers when L-Arginine was given and the nonsmokers (*p* = 0.035).

**Conclusion:**

Short-term smokers showed a reduced response to cold both with and without the administration of L-Arginine. However, absolute MBF values under CPT were lower compared to nonsmokers independently of L-Arginine administration.

## Background

Long-term cigarette smoking is a well-known coronary risk factor that might impair endothelial function [1–6]. Since endothelial dysfunction might be the first step in the cascade towards coronary artery disease, knowledge of the microvascular status of patients with coronary risk factors is of high clinical value [7, 8].

Several studies have suggested the cold pressor test (CPT) as a method to investigate endothelial function [8–10]. This test (semi-)quantitatively assesses myocardial perfusion at rest and with one extremity (hand, foot) stimulated with cold (typically ice-water bath). CPT was performed in combination with different imaging modalities such as positron emission tomography (PET) [1, 10–13], single photon emission computed tomography (SPECT) [14] or magnetic resonance imaging (MRI) [10, 15–19]. Magnetic resonance imaging has been widely used to investigate myocardial perfusion in the past with both a technical [20–25] and also a clinical focus [26–29]. Additionally, endothelium-dependent and endothelium-independent MBF reserve has been investigated via CPT and adenosine stress test in an MRI setting [30–32]. A previous MRI-CPT study also found an impaired endothelial function in patients with type 1 diabetes [16].

Several studies showed that endothelial function is impaired not only after a long smoking career but already in young short-term smokers [10, 13, 17–19].

L-Arginine is the substrate for nitric oxide synthase (NOS) [33, 34] and improved flow response in patients with coronary risk factors [12, 35]. A single publication [12] showed that L-Arginine normalizes coronary vasomotion in long-term smokers. However, effects of L-Arginine on the response to a cold pressor test in young healthy short-term smokers have not been examined, yet.

Hence, the aim of this prospective cohort study was to investigate coronary vasomotion of young healthy short-term smokers via MRI-CPT with and without the administration of L-Arginine and to compare obtained results to the ones from nonsmokers.

## Methods

### Study population

The study was approved by our local Ethics Committee, and written informed consent was obtained from every participant prior to the examination.

We included 10 healthy smokers (5 female/5 male, age: 25.0 ± 2.8 years [mean ± standard deviation], 5.0 ± 2.9 pack years) with no history of cardiac disease. Since this cohort is rather young with a low number of pack years compared to a former PET-study the terms “young” and “short-term smokers” are used throughout the manuscript. The control group consisted of 10 nonsmokers (5 female/5male, age: 34.3 ± 13.6). From all subjects a medical history was obtained and patient files were checked for comorbidities possibly influencing the results of the study. Table 1 summarizes patient characteristics.

**Table 1:** Summary of characteristics

Non-Smokers		Smokers		Pack-years
Sex	Age (years)	Sex	Age (years)	
m	26	m	23	3.0
f	57	m	23	3.5
f	27	f	23	5.0
m	27	m	27	13.0
f	23	f	23	3.0
m	47	f	24	5.0
f	22	f	28	5.5
f	24	m	30	3.5
m	31	f	21	2.5
m	59	m	28	6.0
5f	mean: 34.3	5f	mean: 25.0	5.0
5m	std: 13.6	5m	std: 2.8	2.9

m: male, f: female

### Magnetic resonance imaging

All imaging was performed on a clinical 1.5 T scanner (MAGNETOM SYMPHONY QUANTUM, Siemens Healthcare, Erlangen, Germany) with the use of a 32-channel cardiac array coil (Rapid Biomedical, Rimpar, Germany). First-pass myocardial perfusion imaging was performed using a multislice steady-state free precession sequence acquiring 40 images of three short axis slices over 40 heartbeats. The imaging sequence (repetition time = 2.8 ms, echo time = 1.4 ms, GRAPPA R = 3, flip angle = 50°) provided images with an in-plane resolution of 1.8 × 2.1 mm and a slice thickness of 10 mm. Imaging was done ECG-gated in end-expiratory breath hold to minimize motion artifacts induced by breathing and cardiac motion. For absolute quantification of MBF a prebolus technique [36] with 1 and 4 cc gadolinium based contrast agent (gadolinium-BOPTA) was used for both rest and CPT. Contrast agent was injected at a flow of 4 cc/s followed by a flush of 20 cc of saline to ensure a compact bolus of contrast agent.

An overhead ice-water bath of the left hand with an approximate temperature of 3 °C was used for CPT. The hand was submerged in the ice-water bath for 1 min prior to the start of the CPT measurement to guarantee an adequate stimulus. A staff member controlled the placement of the hand throughout the whole procedure to ensure permanent stimulation. After the CPT scan, a delay of 20 min was inserted to ensure adequate contrast agent wash out and abatement of the stimulus prior to the rest examination.

For the examination with L-Arginine we admitted 14.7 g of L-Arginine-hydrochlorid (Fresenius Kabi Deutschland GmbH, Bad Homburg v.d.H., Germany) in a dilution of 302 ml over 45 min at a flow-rate of 6.7 ml/min using an antecubital i.v. injection before the MR-examination (as suggested in [12]). Both measurements, L-Arginine and non-L-Arginine were performed within a median range of 7 days.

### Data postprocessing

After data acquisition, the images were transferred to a workstation for absolute quantification of myocardial blood flow. The software used was home-written in IDL (interactive data language, Harris Geospatial Solutions, Broomfield, Colorado, United States) and validated in previous studies [26, 37–39]. There, endo- and epicardial contours were drawn manually on every image of the acquired dataset and regions of interest were placed in both blood pools. The myocardium was divided into eight equiangular sectors to evaluate MBF on a regional basis. After averaging signal intensities in the sectors, obtained signal intensity time courses were baseline-corrected and thus converted to courses of relative signal change [40]. A partial volume correction was performed to avoid corruption of the myocardial time courses by the blood pool signal using both blood pool time courses [40]. Data from the ROI in the left ventricle of the 1 cc-bolus served as a basis for the construction of the arterial input function which was constructed separately for every slice. Using the corrected prebolus data and a constraint deconvolution technique (exponential model) MBF was absolutely quantified in cc/g/min in eight sectors on three slices resulting in 24 values per subject and examination as proposed earlier [15, 41]. Since in this study the global endothelial behavior was investigated, regional values were averaged to obtain one value per subject and examination. Sectors containing a visible dark-rim artifact were excluded prior to averaging.

From the data of the rest and CPT examination, a ratio was calculated by dividing the CPT-value by the rest-value.

### Statistical analysis

A Kolmogorov–Smirnov test was used to assess if the obtained MBF data was normally distributed. To compare values obtained from smokers with values from nonsmokers a Mann–Whitney-U-Test for independent samples was used. When comparing rest and CPT of the same patients a Wilcoxon signed-rank test was applied. In all cases *p* < 0.05 was considered statistically significant.

## Results

### Data acquisition

**Fig. 1:**
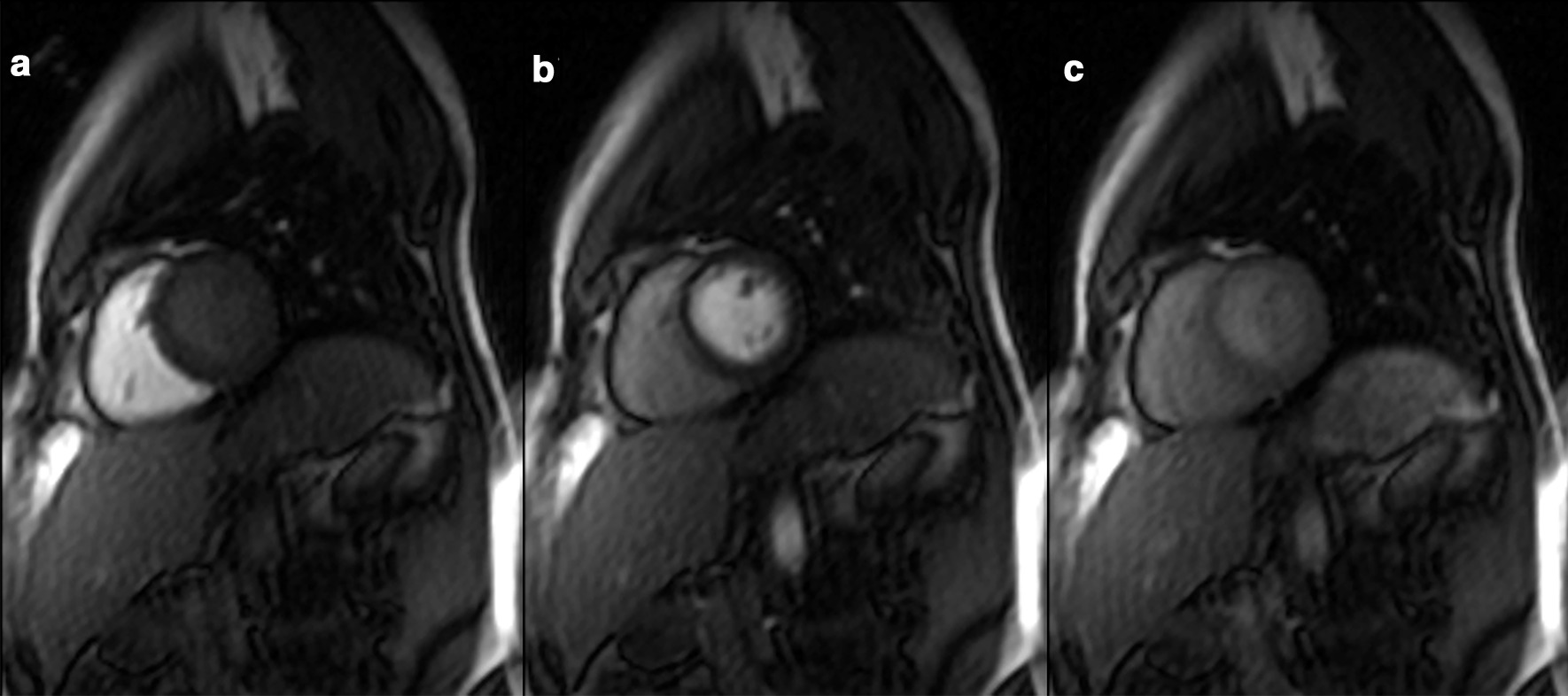
Typical images from a rest examination of a smoker: contrast agent in the right ventricle (**a**), the left ventricle (**b**) and in the myocardium of the left ventricle (**c**)

**Fig. 2:**
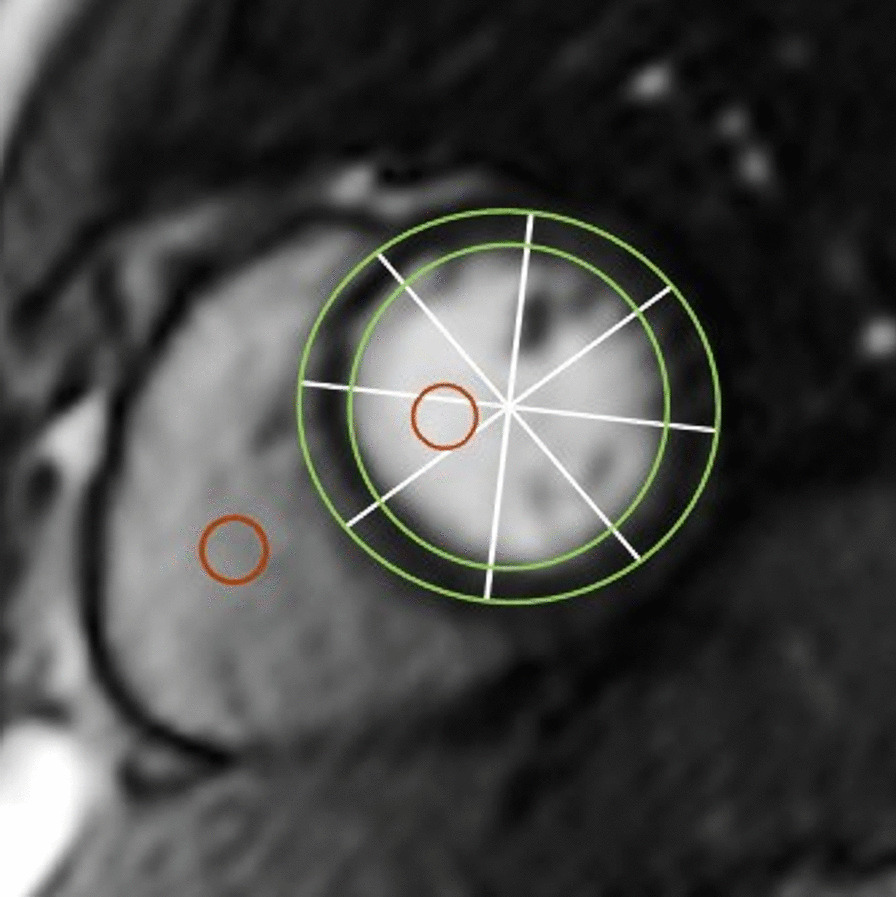
Schematic overview of the image segmentation. Subendo- and subepicardial contours (green) define the leftventricular myocardium which is devided into eight equiangular sectors (white lines). Regions of interest (ROI) were placed in the blood pools for extraction of the arterial input function necessary for the absolute quantification of myocardial blood flow. Blood pool ROIs were also used for partial volume correction

Data could be successfully acquired from every examination. Figure 1 presents typical images of the contrast agent passage of the heart in a rest examination of a smoker. Some participants reported tolerable pain in the left hand during the ice-water bath however, no examination had to be terminated prematurely. Additionally, there was a significant increase of the heart rate during CPT for all groups indicating an adequate stimulus. Quality of the images was sufficient for absolute MBF quantification in all cases. Figure 2 schematically shows the segmentation of one short axis slice.

### Absolute myocardial blood flow values

**Fig. 3:**
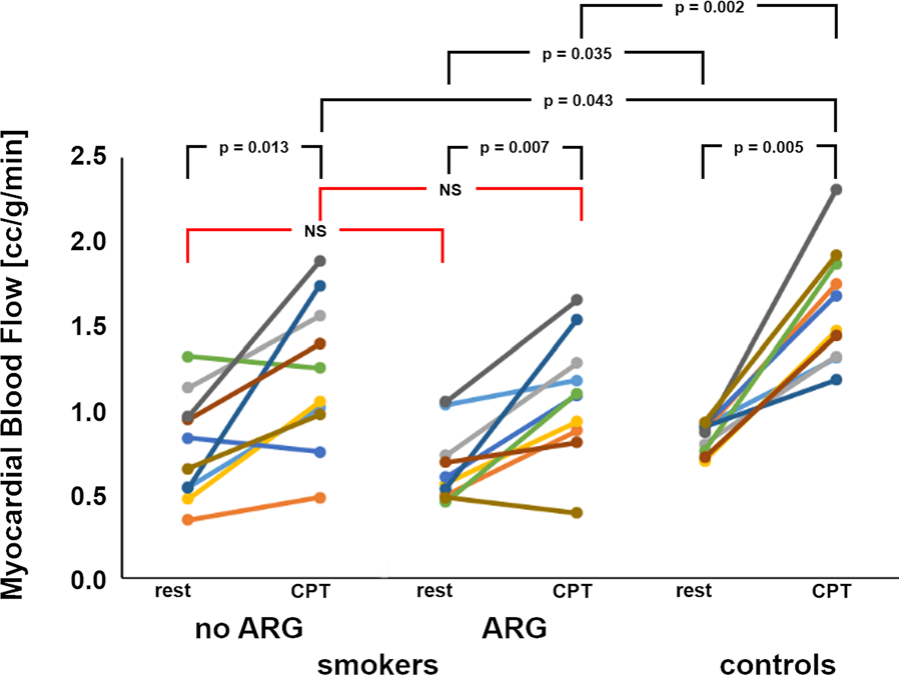
Absolute quantitative myocardial blood flow values for the smokers and the controls. Significant differences are given with respective p-value. no ARG – examination without administration of L-Arginine. ARG – examination with administration of L-Arginine. For better readability, only the two most important non-significant findings are marked in red

Table 2 presents a summary of the obtained MBF values and calculated ratios. Mean MBF for the smokers at rest was 0.77 ± 0.30 cc/g/min and changed to 1.21 ± 0.42 cc/g/min under CPT when no L-Arginine was injected (*p* = 0.013). Administration of L-Arginine delivered values of 0.66 ± 0.21 cc/g/min at rest and 1.09 ± 0.35 cc/g/min under CPT for the smokers (*p* = 0.007). The nonsmokers delivered values of 0.84 ± 0.08 cc/g/min (rest) and 1.63 ± 0.33 cc/g/min (CPT) (*p* = 0.005). Thus, significant differences could be found for all groups between rest and CPT. Moreover, differences between rest (ARG) and rest examination of the nonsmokers (*p* = 0.035), CPT (noARG) and CPT of the nonsmokers (*p* = 0.043) and CPT (ARG) and CPT of the nonsmokers were also significant (*p* = 0.002). Figure 3 presents all values and significant differences. For better visibility, non-significant differences are not shown in Fig. 3. This was the case for rest (noArg) vs. rest (ARG) (*p* = 0.333), CPT (noArg) vs. CPT (ARG) (*p* = 0.333), rest (noARG) vs. rest (nonsmokers) (*p* = 0.684).

**Table 2:** Absolute myocardial perfusion measurements (in cc/g/min) and the ratio calculated as CPT/rest

	Non-smokers			Smokers					
	Rest	CPT	Ratio	Rest(noARG)	CPT(noARG)	Ratio(noARG)	Rest(ARG)	CPT(ARG)	Ratio(ARG)
	0.91	1.31	1.44	0.54	1.02	1.88	1.03	1.18	1.14
	0.88	1.75	1.99	0.35	0.48	1.39	0.49	0.88	1.78
	0.80	1.32	1.65	1.13	1.56	1.37	0.73	1.28	1.75
	0.70	1.47	2.10	0.47	1.05	2.22	0.56	0.93	1.65
	0.91	1.68	1.85	0.83	0.75	0.90	0.60	1.09	1.81
	0.76	1.87	2.46	1.32	1.25	0.95	0.45	1.10	2.42
	0.90	1.18	1.31	0.54	1.74	3.24	0.53	1.54	2.89
	0.72	1.44	2.00	0.95	1.39	1.48	0.69	0.81	1.17
	0.87	2.31	2.66	0.96	1.88	1.96	1.05	1.65	1.57
	0.93	1.92	2.06	0.65	0.97	1.50	0.48	0.39	0.81
mean:	0.84	1.63*	1.95	0.77	1.21*^$^	1.69	0.66^#^	1.09*^§^	1.70
std:	0.08	0.33	0.40	0.30	0.42	0.65	0.21	0.35	0.58

Rest: values under resting conditions; CPT: cold pressor test; Ratio: CPT over rest

^*^p < 0.05 rest vs. CPT; ^#^*p* < 0.05 rest (ARG) vs. Rest (non-smokers); ^$^*p* < 0.05 CPT (noArg) vs. CPT (non-smokers); ^§^*p* < 0.05 CPT (ARG) vs. CPT (non-smokers)

### Ratios

The obtained ratio of the smokers with no L-Arginine was 1.69 ± 0.65 and changed to 1.70 ± 0.58 when L-Arginine was administered. The nonsmokers delivered a ratio of 1.95 ± 0.40. For the ratios no significant differences could be found for any comparison (p > 0.05 in all cases).

## Discussion

The obtained data show a significant increase in MBF after application of CPT for all groups and values are in good accordance with literature even from other modalities [1, 12, 13, 15, 42, 43]. Thus, coronary vasomotion can be studied noninvasively and without the use of ionizing radiation using MRI in a clinical setting.

The presented data show that CPT-MBF of smokers is significantly reduced compared to nonsmokers. This is in accordance to results shown previously [10, 13, 17–19]. Moreover, we also found no significant difference in rest MBF values between smokers and nonsmokers, which is also in accordance with the above mentioned studies. We also found a smaller ratio between MBF-values at rest and CPT in the smokers compared to the nonsmokers. However, in our study this difference was not statistically significant.

Finally, we did not observe a normalization of the MBF-values during acute L-Arginine infusion.

Magnetic resonance imaging bares the opportunity of calculating myocardial blood flow with different approaches. While in [17] and [18] for example phase contrast MRI was used as a basis for flow quantification, in [19] and the present study dynamic contrast enhanced imaging was applied.

Campisi et al. showed that L-Arginine is able to normalize coronary vasomotion in long-term smokers [12]. In our cohort of young short-term smokers, the significant difference of CPT-MBF between smokers and nonsmokers persisted with the administration of L-Arginine. Moreover, the difference of CPT-MBF between smokers with and without L-Arginine was not significant. Additionally, we found no significant correlation between pack years and MBF values or the obtained ratio as Iwado et al. did [10].

The presented study has some limitations, such as the rather small sample size of 10 smokers and 10 non-smokers. However, a power analysis based on earlier results from a MBF/CPT study[39] resulted in exactly that sample size. Since gadolinium-based contrast agent was used in the study, the beforehand calculated sample size was not exceeded due to the well-known possible side effects. Nevertheless, the results might need to be proven in a larger cohort. Since elderly people are supposed to have an already impaired endothelial function the age difference of the investigated cohorts, (34 years vs. 25 years) might have influenced the results. In the present study, the healthy controls were older than the smokers, which might have led to an alignment of the CPT values between the groups. The significant difference between MBF values at rest and under CPT in the control group however, suggests that this had no impact on the results. Another weakness is that absolute MBF-values were not corrected for rate pressure product as done in previous studies [44].

Compared to the study by Campisi et al. in 1999 [12], we examined rather young short-term smokers and thus, observed effects were supposed to be smaller compared to that study. Also compared to Iwado et al. [10] who investigated healthy young smokers in 2002, our cohort was younger (27.4 ± 4.4 vs. 25.0 ± 2.8 years) with even a greater discrepancy in pack years (9.4 ± 4.9 (Iwado et al.) vs. 5.0 ± 2.9 (this study)). These aspects might explain the smaller differences observed in our study since our cohort might have been very early in the onset of endothelial dysfunction.

The presented study did not reproduce the effect of L-Arginine on the MBF-values as shown in a former PET-study (12), i.e. coronary vasomotion was not normalized by L-Arginine. This might be explained by the difference in the cohorts for instance long-term smokers vs. short-term smokers, smoking habits and age. Moreover, effects of short-term smoking on the endothelium might just be strong enough to lower the response during CPT. However, since the differences are rather small, other effects like a too low sensitivity of the applied technique might have masked the neutralization via L-Arginine.

Since results were not compared to other modalities like PET and due to the admittedly limited sample size, findings have to be reproduced in larger cohorts with an improved age-matching to further prove generalizability.

## Conclusions

In conclusion, the presented data point towards the hypothesis that in young smokers with only a small number of pack years endothelial function might already be impaired. However, acute administration of L-Arginine did not normalize flow response during stimulation with cold as shown earlier in our, admittedly, small cohort. Thus, the presented results might need further support by larger studies. Finally, the study is an additional example for the benefit of absolute quantitative numbers obtained from MRI data. The shown differences in MBF-values would not have been revealed by a pure visual inspection of the perfusion datasets.

## Data Availability

The datasets generated during and/or analyzed during the current study are available from the corresponding author on reasonable request.

## Abbreviations

MBF: Myocardial blood flow
CPT: Cold pressor test
MRI: Magentic resonance imaging
PET: Positron emission tomography
SPECT: Single photon emission computed tomography
NOS: Nitric oxide synthase
ECG: Electrocardiogram
i.v.: Intravenous

## References

[1] Campisi R, Czernin J, Schöder H, et al.: Effects of long-term smoking on myocardial blood flow, coronary vasomotion, and vasodilator capacity. *Circulation* 1998:119–125. 10.1161/01.cir.98.2.119 9679717

[2] Gopal DM, Kalogeropoulos AP, Georgiopoulou VV (2012). Cigarette smoking exposure and heart failure risk in older adults : the health, aging, and body composition study. Am Heart J.

[3] Ahmed AA, Patel K, Nyaku MA (2015). Risk of heart failure and death after prolonged smoking cessation role of amount and duration of prior smoking. Circ Hear Fail.

[4] Kamimura D, Cain L, Mentz R, et al.: Cigarette smoking and incident heart failure. 2018:2572–2582. 10.1161/CIRCULATIONAHA.117.031912 PMC6085757 29661945

[5] Zeiher AM, Schächinger V, Minners J (1995). Long-term cigarette smoking impairs endothelium-dependent coronary arterial vasodilator function. Circulation.

[6] Selwyn AP, Kinlay S, Creager M, Libby P, Ganz P (1997). Cell dysfunction in atherosclerosis and the ischemic manifestations of coronary artery disease. Am J Cardiol.

[7] Sanz J, Fayad ZA (2008). Imaging of atherosclerotic cardiovascular disease. Nature.

[8] Deanfield JE, Halcox JP, Rabelink TJ (2007). Endothelial function and dysfunction testing and clinical relevance. Circulation.

[9] Schindler TH, Zhang X, Prior JO (2007). Assessment of intra- and interobserver reproducibility of rest and cold pressor test-stimulated myocardial blood flow with 13N-ammonia and PET. Eur J Nucl Med Mol Imaging.

[10] Iwado Y, Yoshinaga K, Furuyama H (2002). Decreased endothelium-dependent coronary vasomotion in healthy young smokers. Eur J Nucl Med.

[11] Schindler TH, Facta AD, Prior JO (2006). PET-measured heterogeneity in longitudinal myocardial blood flow in response to sympathetic and pharmacologic stress as a non-invasive probe of epicardial vasomotor dysfunction. Eur J Nucl Med Mol Imaging.

[12] Campisi R, Czernin J, Scho H, Sayre JW, Schelbert HR (1999). L-arginine normalizes coronary vasomotion in long-term smokers. Circulation.

[13] Ochi N, Yoshinaga K, Ito YM, et al.: Comprehensive assessment of impaired peripheral and coronary artery endothelial functions in smokers using brachial artery ultrasound and oxygen-15-labeled water PET. *J Cardiol* 2016. 10.1016/j.jjcc.2015.10.006 26620846

[14] Cicala S, Pellegrino T, Storto G (2010). Noninvasive quantification of coronary endothelial function by SPECT imaging in children with a history of Kawasaki disease. Eur J Nucl Med Mol Imaging.

[15] Ritter CO, Kowalski M, Weng AM, Beer M, Hahn D, Köstler H (2012). Quantitative myocardial perfusion imaging with a MR cold pressor test. Magn Reson Med.

[16] Weng AM, Wilimsky S, Bender G, Hahner S, Köstler H, Ritter CO: Magnetic resonance cold pressor test to investigate potential endothelial dysfunction in patients suffering from type 1 diabetes. *J Magn Reson Imaging* 2018:1595–1601. 10.1002/jmri.26191 29897641

[17] Ichikawa Y, Kitagawa K, Kato S, et al.: Altered coronary endothelial function in young smokers detected by magnetic resonance assessment of myocardial blood flow during the cold pressor test. Int J Cardiovasc Imaging 2014. 10.1007/s10554-014-0387-y 24519431

[18] Kato S, Kitagawa K, Yoon YE, et al.: Detection of diminished response to cold pressor test in smokers: assessment using phase-contrast cine magnetic resonance imaging of the coronary sinus. Magn Reson Imaging 2014. 10.1016/j.mri.2013.12.015 24480156

[19] Fairbairn TA, Motwani M, Mather AN (2014). Cardiac MR imaging to measure myocardial blood flow response to the cold pressor test in healthy smokers and nonsmokers. Radiology.

[20] Köstler H, Sandstede JW, Lipke C (2003). Auto-Sense perfusion imaging of the whole human heart. J Magn Reson Imaging.

[21] Ritter CO, Wilke A, Wichmann T, Beer M, Hahn D, Köstler H (2011). Comparison of intravascular and extracellular contrast media for absolute quantification of myocardial rest-perfusion using high-resolution MRI. J Magn Reson Imaging.

[22] Stäb D, Wech T, Breuer FA (2014). High resolution myocardial first-pass perfusion imaging with extended anatomic coverage. J Magn Reson Imaging.

[23] Otazo R, Kim D, Axel L, Sodickson DK (2010). Combination of compressed sensing and parallel imaging for highly accelerated first-pass cardiac perfusion MRI. Magn Reson Med.

[24] Knott KD, Camaioni C, Ramasamy A, et al.: Quantitative myocardial perfusion in coronary artery disease: a perfusion mapping study. J Magn Reson Imaging 2019. 10.1002/jmri.26668 PMC6767569 30684288

[25] Nakajima T, Oriuchi N, Tsushima Y, Funabasama S, Aoki J, Endo K (2004). Noninvasive determination of regional myocardial perfusion with first-pass magnetic resonance (MR) imaging. Acad Radiol.

[26] Weng AM, Ritter CO, Beer M, Hahn D, Köstler H (2014). Quantitative pixelwise myocardial perfusion maps from first-pass perfusion MRI. Br J Radiol.

[27] Vogel-claussen J, Skrok J, Shehata ML (2011). Right and left ventricular myocardial perfusion reserves correlate with right ventricular function and pulmonary hemodynamics in patients with pulmonary arterial hypertension. Radiology.

[28] Unlu M, Anik Y, Demirci A, Ural D, Kahraman G, Komsuoglu B (2006). Cardiac MRI in ischemic heart disease with severe coronary artery stenosis. Acad Radiol.

[29] Ma H, Zhang Y, Chen J, Yang J (2019). Whole left ventricular coverage versus conventional 3-slice myocardial perfusion magnetic resonance imaging for the detection of suspected coronary artery disease. Acad Radiol.

[30] Cullen JH, Horsfield MA, Reek CR, Cherryman GR, Barnett DB, Samani NJ (1999). A myocardial perfusion reserve index in humans using first-pass contrast-enhanced magnetic resonance imaging. J Am Coll Cardiol.

[31] Pack NA, DiBella EVR (2010). Comparison of myocardial perfusion estimates from dynamic contrast-enhanced magnetic resonance imaging with four quantitative analysis methods. Magn Reson Med.

[32] Fritz-Hansen T, Hove JD, Kofoed KF, Kelbaek H, Larsson HBW (2008). Quantification of MRI measured myocardial perfusion reserve in healthy humans: a comparison with positron emission tomography. J Magn Reson Imaging.

[33] Morris SM (2005). Arginine metabolism in vascular biology and disease. Vasc Med.

[34] Michel T (2013). R is for arginine : metabolism of arginine takes off again, in new directions. Circulation.

[35] Drexler H, Zeiher AM, Meinzer K, Just H (1991). Correction of endothelial dysfunction in coronary microcirculation of hypercholesterolaemic patients by L-arginine. Lancet.

[36] Köstler H, Ritter CO, Lipp M, Beer M, Hahn D, Sandstede J (2004). Prebolus quantitative MR heart perfusion imaging. Magn Reson Med.

[37] Weng AM, Ritter CO, Lotz J, Beer MJ, Hahn D, Köstler H (2010). Automatic postprocessing for the assessment of quantitative human myocardial perfusion using MRI. Eur Radiol.

[38] Köstler H, Ritter C, Lipp M, Beer M, Hahn D, Sandstede J (2008). Comparison of different contrast agents and doses for quantitative MR myocardial perfusion imaging. J Magn Reson Imaging.

[39] Weng AM, Wilimsky S, Bender G, Hahner S, Köstler H, Ritter CO (2018). Magnetic resonance cold pressor test to investigate potential endothelial dysfunction in patients suffering from type 1 diabetes. J Magn Reson Imaging.

[40] Köstler H, Ritter CO, Reiss-Zimmermann M, Beer M, Hahn D, Sandstede J (2004). Correction for partial volume errors in MR heart perfusion imaging. Magn Reson Med.

[41] Weng AM, Ritter CO, Lotz J, Beer M, Hahn D, Köstler H (2010). Automatic postprocessing for the assessment of quantitative human myocardial perfusion using MRI. Eur Radiol.

[42] Wang Y, Passic M (2013). Preliminary quantitative myocardial perfusion in response to cold pressor test in normals. J Cardiovasc Magn Res.

[43] Prior JO, Schindler TH, Facta AD (2007). Determinants of myocardial blood flow response to cold pressor testing and pharmacologic vasodilation in healthy humans. Eur J Nucl Med Mol Imaging.

[44] Williams MC, Mirsadraee S, Dweck MR (2017). Computed tomography myocardial perfusion vs 15O-water positron emission tomography and fractional flow reserve. Eur Radiol.

